# Plasma Optimization as a Novel Tool to Explore Plant–Microbe Interactions in Climate Smart Agriculture

**DOI:** 10.3390/microorganisms13010146

**Published:** 2025-01-13

**Authors:** Binoop Mohan, Chandrima Karthik, Doni Thingujam, Karolina M. Pajerowska-Mukhtar, Vinoy Thomas, M Shahid Mukhtar

**Affiliations:** 1 Department of Biology, University of Alabama at Birmingham (UAB), 3100 East Science Hall, 902 14th Street South, Birmingham, AL 35294, USA; binoopm2@uab.edu; 2 Department of Mechanical and Materials Engineering, University of Alabama at Birmingham (UAB), Birmingham, AL 35294, USA; ckarthik@uab.edu (C.K.); vthomas@uab.edu (V.T.); 3 Department of Biological Sciences, Clemson University, 132 Long Hall, Clemson, SC 29634, USA; dthingu@clemson.edu (D.T.); kmukhta@clemson.edu (K.M.P.-M.); 4 Biosystems Research Complex, Department of Genetics & Biochemistry, Clemson University, 105 Collings St., Clemson, SC 29634, USA

**Keywords:** plasma optimization, plant–microbe interaction, plasmidome, transcriptome, metabolome

## Abstract

Plasma treatment has emerged as a promising tool for manipulating plant microbiomes and metabolites. This review explores the diverse applications and effects of plasma on these biological systems. It is hypothesized that plasma treatment will not induce substantial changes in the composition of plant microbiomes or the concentration of plant metabolites. We delve into the mechanisms by which plasma can regulate microbial communities, enhance antimicrobial activity, and recruit beneficial microbes to mitigate stress. Furthermore, we discuss the optimization of plasma parameters for effective microbiome interaction and the role of plasmids in plant–microbe interactions. By characterizing plasmidome responses to plasma exposure and investigating transcriptional and metabolomic shifts, we provide insights into the potential of plasma as a tool for engineering beneficial plant–microbe interactions. The review presented herein demonstrates that plasma treatment induces substantial changes in both microbial community composition and metabolite levels, thereby refuting our initial hypothesis. Finally, we integrate plasmidome, transcriptome, and metabolome data to develop a comprehensive understanding of plasma’s effects on plant biology and explore future perspectives for agricultural applications.

## 1. Introduction

Plasma is generally an ionized gas with a wide range of applications across various fields, including industry [1], medicine [2], energy [3], environment [4], agriculture [5], and space research [6]. Researchers worldwide are currently exploring innovative ways to harness the potential for advanced applications. One of the most intriguing aspects of plasma is its role in agriculture, which has proven effective in enhancing seed germination, improving plant growth, and preservation [5]. Plasma also shows promise in influencing the plant microbiome and enhancing plant resistance by activating plant metabolites [7,8]. This review will explore different types of plasma that are beneficial in various surface treatments and understand the role of plasma in regulating plant–microbe interactions. The optimization of plasma will be crucial to establish unique treatment strategies to aid plant-related research. Recent findings indicate plasma treatment significantly influences plasmidome diversity, expression patterns, and metabolite production [9]. These factors are crucial in determining the microbiome diversity associated with plants. We will also focus on unique and novel applications related to the plant microbiome, demonstrating how plasma optimization can be effectively used in the future to improve plant resilience [7,9]. Additionally, plasma technology has shown potential in pest management, soil improvement, and maintaining agriculture product quality. Non-thermal plasma (NTP) and plasma-activated water (PAW) have been particularly noted for their benefits in pre-harvest and post-harvest processes. By harnessing these technologies, we can develop sustainable agricultural practices that enhance crop yields and resilience against environmental stresses [9].

## 2. Plasma Treatment Systems: A Typology for Phytological Research

**Figure 1:**
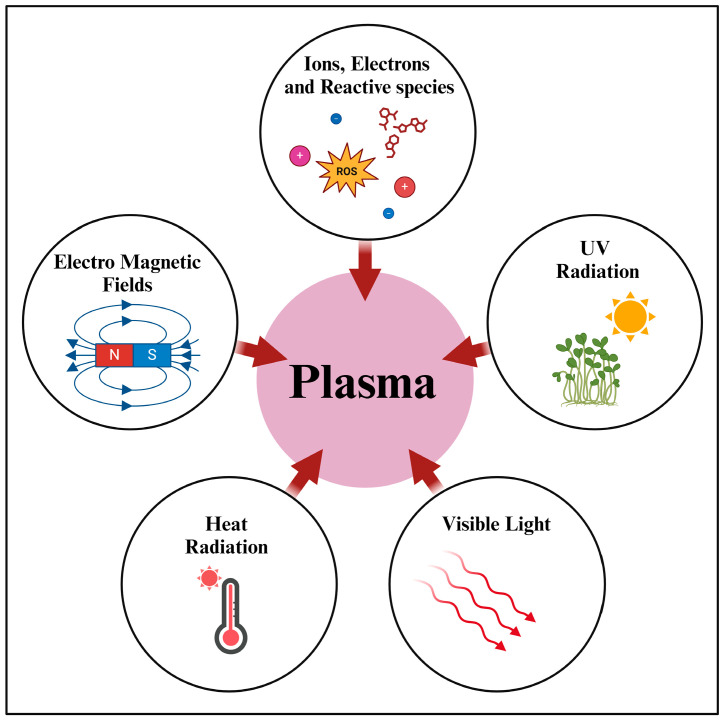
Comprehensive diagram of the fundamental components in a plasma state.

Plasma technology has emerged as a promising tool in agricultural research and applications, offering innovative solutions in a broad spectrum of areas to enhance crop productivity, improve food safety, and promote sustainable farming practices. Plasma features a mix of electrons, ions, and neutral species capable of inducing significant chemical and physical changes in treated materials through complex interactions (Figure 1) [10]. Agricultural plasma treatments generate reactive species like hydroxyl radicals, atomic oxygen, ozone, and nitrogen oxides. These species can damage cellular components through oxidative mechanisms, such as lipid peroxidation and protein oxidation [11]. Plasma systems used in research can be broadly classified into two main categories: thermal plasma (TP) and NTP systems. TP systems are exemplified by extremely high temperatures, characteristically surpassing 10,000 °C. In thermal plasma, all particles are in a thermal steady state. While effective for certain industrial processes, thermal plasma has a confined scope in phytological research due to its potential to cause thermal damage to heat-sensitive plant materials. NTP, otherwise known as cold atmospheric plasma (CAP) or low-temperature plasma (LTP), operating at or near room temperature, is highly advantageous for botanical research [12]. The largest concentration of research and development is found in the Asia-Pacific region, particularly in Japan, China, and South Korea, accounting for approximately 45% of global plasma agriculture research activities [7]. The global market for agricultural plasma technologies is estimated at 100–150 million USD, with an annual growth rate of 15–20% [7,13]. The technology’s applications are distributed across seed treatment (40%), post-harvest processing (30%), soil treatment (20%), and water treatment (10%), with ongoing research focused on optimizing these processes for commercial viability [7].

### 2.1. Dielectric Barrier Discharge (DBD) Plasma System

**Figure 2:**
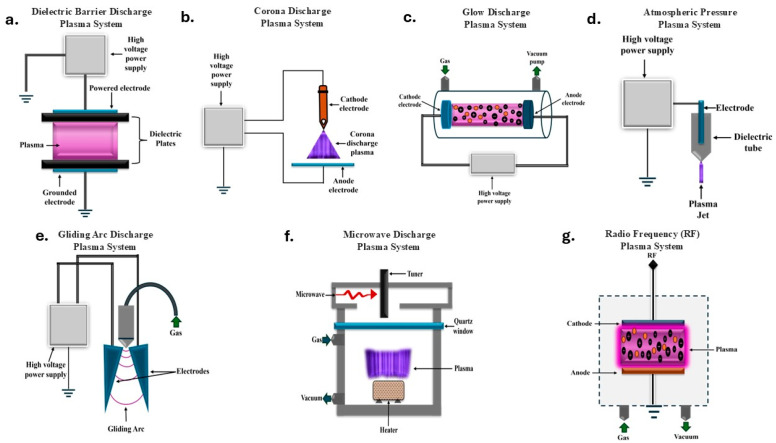
Illustration of different types of plasma and a schematic representation of (**a**) a dielectric barrier discharge plasma system, (**b**) corona discharge plasma system, (**c**) glow discharge plasma system, (**d**) atmospheric pressure plasma system, (**e**) microwave discharge plasma system, and (**f**) radio frequency plasma system, (**g**) Radio Frequency (RF) Plasma System.

DBD plasma systems comprise two electrodes separated by one or more dielectric barriers. When high voltage is applied, it creates micro-discharges across the gap, generating LTP (Figure 2a). DBD systems operate at atmospheric pressure, consume low power, and produce homogeneous plasma. They generate various reactive species, making them effective for seed treatment, disinfestation, and surface modification [14]. In addition to surface disinfection, plasma technology effectively treats bioaerosols, offering comprehensive pathogen control in agricultural environments [15]. In agriculture, DBD plasma has displayed favorable outcomes in enhancing seed germination, improving plant growth, and increasing crop yields. For example, DBD plasma treatment increased wheat seed germination potential by 6% and germination potential by 6.7% compared to the control while improving plant height, root length, and fresh weight at the seedling stage [5]. The ability to operate at atmospheric pressure and generate a wide range of reactive species makes DBD plasma systems particularly suitable for large-scale applications [16].

### 2.2. Corona Discharge (CD) Plasma System

CD plasma systems employ a sharp electrode tip and a ground electrode to create a diffuse plasma formation, as shown in Figure 2b. This configuration generates a variable electric field, resulting in biased ionization of the surrounding gas. Corona discharge plasmas operate at atmospheric pressure and produce various reactive species, including ozone, which is significantly efficacious for decontamination and sterilization purposes [7]. In agricultural applications, CD plasma has been used for seed treatment, improving germination rates and seedling growth. The potential to generate ozone makes it particularly useful in agriculture for post-harvest treatments, extending the shelf life of fruits and vegetables by reducing microbial contamination. CD plasma has also shown potential in enhancing plant nutrient assimilation in plants and modifying seed surfaces to improve water absorption. The mildly intricate design and low power requirements of corona discharge systems make them attractive for small-scale treatments.

### 2.3. Glow Discharge (GD) Plasma System

GD plasma systems operate at low pressures and harness a continuous electrical discharge between two electrodes (see Figure 2c). This system produces a stable, uniform plasma with distinct domains of light emission. GD plasma systems generate a high concentration of reactive species and are particularly effective for surface modifications [17]. In agricultural applications, GD plasma has been used for seed treatment, enhancing germination rates and early seedling growth. For instance, the treatment of *Raphanus sativus* L. (radish) seeds with low-pressure GD plasma at 20 Pa pressure yielded a maximum seedling length [5]. GD plasma can also be used to modify soil properties, potentially improving nutrient bioavailability and water retention. The uniform distribution of GD plasma makes it suitable for treating large batches of seeds or agricultural products simultaneously. However, the low-pressure requirement can constrain its implementation in field conditions, making it more appropriate for controlled environments or industrial-scale treatments [18,19].

### 2.4. Atmospheric Pressure Plasma (APP) System

APP systems operate at barometric pressure, obviating the necessity of vacuum equipment (see Figure 2d). These systems can produce non-thermal plasmas using diverse configurations, including plasma jets, corona discharges, and dielectric barrier discharges. APP systems produce a wide range of reactive species, including reactive oxygen and nitrogen species (RONS), making them versatile for phytological studies [7]. In agriculture, APP has been used for seed treatment, enhancing germination rates, seedling growth, and stress tolerance. For example, the APP treatment of *Pisum sativum* L. (pea) seeds for 30 and 60 s increased the sprouting rates to 80% and 74%, respectively, compared to 40% in the control samples [5].

### 2.5. Gliding Arc Discharge (GAD) Plasma System

GAD plasma systems utilize two diverging electrodes to initiate a plasma arc that advances along the electrode length; this configuration yields a non-equilibrium plasma with thermal and non-thermal characteristics (see Figure 2e). GAD plasmas operate at atmospheric pressure and can generate a high concentration of reactive species, including nitrogen oxides and ozone [20,21]. GAD plasma has shown potential for water treatment, soil remediation, and seed treatment in agriculture. For instance, a study using air GAD plasma for seawater sterilization demonstrated complete inactivation of *Vibrio parahaemolyticus* in a 5000 mL volume within 20 min without significantly impacting the key water quality parameters [20].

### 2.6. Microwave Discharge (MD) Plasma System

MD plasma systems harness electromagnetic waves in the GHz frequency spectrum to induce, stabilize, and retain plasma (see Figure 2f). These systems can operate at multiple pressures, from low to barometric, and generate high-density plasmas with an elevated degree of ionization [17,22]. The high-energy nature of microwave plasmas makes them particularly effective for surface modifications of seeds, potentially improving water absorption and nutrient uptake. In phytobiological research, microwave plasma has been used for seed treatment, enhancing germination rates and seedling growth. These systems have also shown potential for degrading pesticides and other organic pollutants in water and soil. The ability to generate high-density plasmas with a broad spectrum of reactive species makes these systems suitable for various agricultural applications, such as seed treatment and environmental remediation [23].

### 2.7. Radio Frequency (RF) Plasma System

RF plasma systems operate in the MHz frequency range and can generate plasmas at various pressures, from low to atmospheric (see Figure 2g). RF plasma systems produce homogeneous, steady-state plasmas with a substantial degree of control over various plasma parameters, such as gas flow, treatment time, etc. RF plasmas generate a wide range of reactive species and are particularly effective in thin-film deposition and surface alterations [17,24]. In plants, RF plasma has been used for seed treatment and the modification of agricultural materials. For example, treating wheat seeds with low-pressure RF plasma at 80 W power resulted in notable progress in seed germination potential, germination rate, and subsequent plant maturation [5,25]. RF plasma systems have also shown potential in the modification of soil properties and the degradation of organic pollutants. The high degree of control over plasma parameters in RF systems allows for the fine-tuning of treatments for various bio applications.

## 3. Impacts of Plasma Treatment on Plant Microbiomes and Metabolites

**Figure 3:**
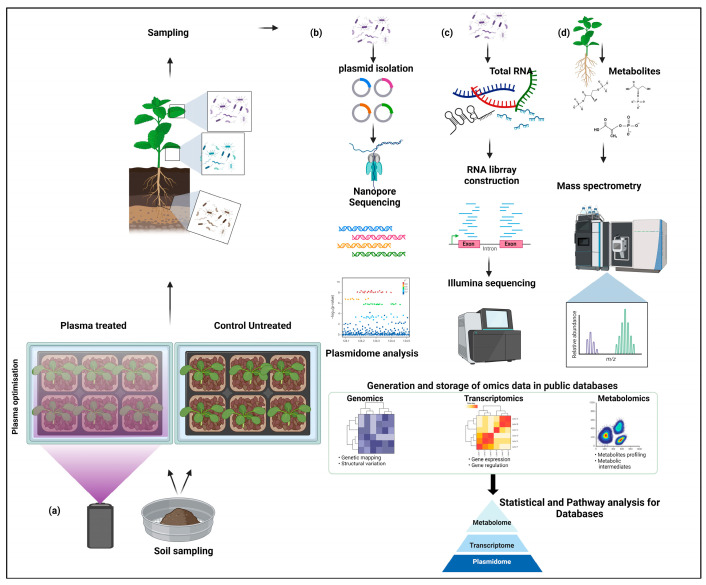
Illustration of the plasma optimization of environmental soil samples followed by (**a**) the sampling of treated and control samples, (**b**) the isolation of plasmidome from the microbial sample, (**c**) the transcriptome profiling to quantify the differentials expression, and (**d**) metabolite isolation and profiling, followed by an interactome study combining plasmidome, transcriptome, and metabolome data.

Plasma technology, which harnesses the unique properties of ionized gases, has emerged as a promising approach in agricultural research, offering the potential to modulate the complex microbial communities that reside on plant surfaces, as well as the diverse array of metabolites they produce [26,27]. The plant microbiomes harbor a vibrant and diverse array of beneficial microbes that can profoundly influence various aspects of plant functioning, from growth and development to their interactions with the surrounding environment [28,29]. These epiphytic microbial communities are subject to a range of environmental stressors, and mounting evidence suggests that their composition and activities can have far-reaching impacts on plant fitness [30]. Intriguingly, recent studies have demonstrated that plasma treatment can significantly alter the composition of microbiomes, potentially through the direct effects of reactive oxygen and nitrogen species generated by the plasma on microbial cells or indirectly by modifying the physicochemical properties of plant surfaces [28,29,31]. The resulting shifts in microbial community structure have been linked to changes in the production of secondary metabolites, some of which may have beneficial effects on plant growth, defense, or stress tolerance. As the field of plasma agriculture continues to evolve, a deeper understanding of the intricate relationships between plasma-induced modifications to the microbiome and the resulting changes in plant metabolite profiles can be crucial (see Figure 3). While plasma technology offers many benefits in agriculture, it is important to recognize both its potential negative impacts on crops and its applications in weed control. Recent studies have indicated that excessive plasma treatment can reduce crop yields by 15–30% due to physiological disruptions, especially during sensitive growth stages. However, these same properties make plasma treatment a promising tool for weed control, with research showing its effectiveness in both pre-emergence applications through soil surface sterilization and post-emergence treatments that directly damage weed foliage [32].

### 3.1. Regulating Microbial Community Through Plasma Treatment

Recent advancements in plasma technology have significantly influenced plant microbiomes in various ways. A notable impact is the enhancement of microbial activity, which boosts plant resilience and growth through non-thermal atmospheric plasma treatment, particularly in rice (see Figure 3a) [33]. Besides promoting overall plant growth, plasma treatment induces major shifts in plant–microbe interactions, including increased microbial colonization, improved pathogen resistance, and elevated levels of phytohormones [34,35]. Plasma treatment plays a crucial role in regulating the microbiome by generating reactive species that selectively affect beneficial and pathogenic microbes. Plasma treatment’s differential effects on pathogenic versus beneficial microorganisms result from several key biological and structural factors [36]. Beneficial soil microorganisms often demonstrate higher survival rates due to evolved stress resistance mechanisms, including enhanced antioxidant systems and DNA repair capabilities. Studies have shown that beneficial rhizobacteria can maintain 60–70% survival under conditions that eliminate 90% of pathogens [7]. This resistance is attributed to structural advantages such as thicker peptidoglycan layers and more rigid membrane structures in beneficial bacteria, along with their natural adaptation to oxidative stress in soil environments [37]. However, optimizing plasma conditions to target specific plant microbiomes remains critical. For instance, a recent study demonstrated that plasma-processed air treatment in lettuce significantly reduced the microbial content without affecting the quality of lettuce [38]. This innovative approach, using air as a plasma source, can minimize water usage for cleaning lettuce and profoundly impact surface microbes [39].

### 3.2. Role in Antimicrobial Activity

Plasma treatment has emerged as a promising approach to enhance the antimicrobial properties of plants by modulating their metabolome and metabolites [40]. The generation of plasma creates a rich mixture of highly reactive oxygen and nitrogen species, such as hydroxyl radicals, superoxide radicals, and hydrogen peroxide, all of which possess strong oxidative capabilities [41]. The strong effect of the reactive species creates oxidative stress that can damage the cell membrane and cause DNA damage and can effectively be applied to multidrug-resistant pathogens [42]. Research has shown that NTP can significantly reduce the viability of plant pathogenic bacteria over time [43,44,45,46]. For example, GAD has been effective inactivating *Erwinia carotovora*, a pathogen affecting potatoes, by disrupting its bacterial membrane. Additionally, plasma treatments have been found to slow down the growth and reproduction of *Clavibacter michiganensis* and *Erwinia amylovora* [44,47]. More recent studies have highlighted that direct current (DC)-based GD plasma can quickly eliminate several plant pathogens, including *Clavibacter michiganensis* subsp. *sepedonicus*, *Dickeya solani*, *Xanthomonas campestris* pv. *campestris*, *Pectobacterium atrosepticum*, and *Pectobacterium carotovorum* subsp. *carotovorum* [46,48].

### 3.3. Recruiting Beneficial Microbes to Combat Stress

Plasma treatment can induce systemic resistance in plants, enhancing their immune responses and making them more resilient to future infections [49]. This dual action not only helps in controlling plant diseases but also supports a balanced microbial ecosystem, which is crucial for plant health and growth [50]. While the reactive species can influence beneficial soil microbes, studies suggest that plasma treatment can selectively target pathogens with minimal negative effects on beneficial microbes like plant growth-promoting rhizobacteria (PGPR), depending on the treatment parameters [51]. By reducing the need for chemical pesticides, plasma treatment offers a sustainable and eco-friendly approach to managing plant health [52]. Plasma technology provides an alternative to chemical additives for pathogen control, but it necessitates energy input for operation. Energy consumption varies depending on the application; air treatment systems generally require 0.1–0.2 kWh per 1000 cubic meters, whereas water treatment systems typically consume 0.4–0.8 kWh per cubic meter [53]. Techniques like pulsed operation and smart control systems can optimize energy use, making plasma technology potentially more efficient than traditional chemical treatments despite the initial energy requirements. Plasma treatment has been shown to significantly enhance the growth of plant growth-promoting bacteria *Klebsiella pneumoniae* while preserving the integrity of its cell membranes [54,55].

### 3.4. Role in Metabolite Production

The influence of plasma treatment on plant metabolites can be attributed to the activation of specific signaling pathways and transcriptional regulation. These reactive species can induce various biochemical and molecular changes in plants, ultimately leading to the production of specialized metabolites with enhanced antimicrobial activities [56]. Several studies have shown that plasma treatment can significantly increase the accumulation of secondary metabolites, such as phenolic compounds, flavonoids, and terpenes, in medicinal plants [57]. These metabolites are known to exhibit a wide range of antimicrobial properties, including the ability to disrupt microbial cell membranes, inhibit enzyme activities, and interfere with nutrient uptake. Furthermore, plasma treatment has been found to enhance the production of antimicrobial compounds like ursolic acid. Bacteria treated with plasma can also increase the production of salicylic acid levels in rice plants, which boosts their growth and improves their resistance to diseases [58].

## 4. Optimization of the Plasma Parameters for Microbiome Interactions

Optimizing plasma parameters for microbiome interactions in phytology is a complex process that requires careful consideration of diverse elements. The impact of plasma treatment goes beyond direct cellular interactions, affecting genetic elements such as plasmids. These plasmids are essential for microbial adaptation, antimicrobial resistance, and the dynamics of plant–microbe relationships. The goal is to maximize the beneficial effects on plant growth and pathogen control while ensuring plant safety and treatment efficiency. Treatment parameters such as plasma treatment duration, applied voltage, gas composition, and distance from the plasma source significantly influence the effectiveness of the treatment [59]. The optimal duration of treatment varies according to the application; for instance, bacterial inactivation may be effective in as little as two to three minutes [60]. The time required for plasma to inactivate bacteria varies significantly between species. For example, Pseudomonas species can be inactivated in as little as 1 min, whereas Bacillus species may need up to 30 min. This difference underscores the need to consider species-specific traits when developing plasma-based decontamination methods [61,62]. The generation of specific reactive species is crucial for attaining the desired agricultural effects. RONS play important roles in plant growth stimulation, pathogen control, and stress tolerance [7,41]. The pH alteration resulting from plasma treatment also affects both antimicrobial activity and plant responses [63]. LTP exposure can lead to acidic conditions in treated solutions. For instance, a study found that low-temperature plasma treatment of deionized water resulted in a significant decrease in pH, creating an acidic environment [64].

Use case optimization is necessary for different uses such as seed treatment, plant growth promotion, pathogen control, and abiotic stress tolerance [65]. For instance, plasma treatment of sunflower seeds has been shown to stimulate root and lateral organ growth by 9–14% compared to untreated control plants [66]. Adjusting the power of the plasma can control the intensity and penetration depth of these reactive species, optimizing the microbial stimulation without causing damage. Different plasma types produce varying reactive species at different rates and intensities. For instance, DBD plasma generates a high concentration of RONS, which can enhance microbial diversity and activity by creating a more favorable environment for beneficial microbes [67]. The type of gas used in plasma generation (e.g., nitrogen, argon, air, etc.) affects the types and concentrations of reactive species produced [68]. The research has explored using plasma technology to generate dinitrogen pentoxide from air, which forms nitric acid in water and dissociates into nitrate, providing an alternative to traditional nitrogen fertilizers by directly incorporating plasma-activated nitrogen into plant nutrition [69]. Tailoring the gas composition to the specific needs of the microbiome can optimize these interactions. Challenges in optimization include the standardization of protocols, scalability from laboratory to field applications, accounting for species-specific responses, and evaluating long-term effects on plant health and crop yield.

## 5. Plasmid Associated with Plants

Plasma-induced environmental changes impact not only immediate microbial responses but also the genetic adaptability of microorganisms, particularly through plasmids. Microbial diversity is not limited to the associated host, but it is being influenced and shaped upon as an evolutionary process to adapt to the environmental stimulus. Clues to the stress-related response can be explored through identifying the overall plasmidome (complete plasmid present in a population sample) associated with the plant [70]. Plasma treatment can have a strong influence on the plant microbiome, and it can also regulate the plasmid content of the associated microbes. Antimicrobial resistance, pathogenicity, and the influence of beneficial microbes are all influenced through plasmids, and thus, it will be interesting to study the impact that plasma treatment can have on the plasmidome [71]. Recognizing how plasma treatment affects both immediate physiological responses and long-term genetic adaptations via plasmids is essential for creating effective and sustainable agricultural practices.

### 5.1. Plasmids in Plant Pathogens

Plant pathogens carry a wide range of plasmids often involved in virulence function, and the transfer of genes can impart virulence to other associated microbes [72]. It can be anticipated that plasmids play a regulatory role in plant–pathogen interactions, but understanding the molecular mechanism needs to be explored further. On the other hand, *Rhodococcus*-related virulence in plants is regulated by FasR, which is a plasmid-associated gene that plays a crucial role in the cytokinin biosynthesis pathway. This virulence model clearly indicates the regulatory role of a plasmid-associated gene to the chromosomal gene activation that leads to virulence in plants [73,74]. Antibiotic-resistant genes are a major player when it comes to host–microbe interactions. A wide range of hosts, including humans, plants, and animals, are under threat due to the rising number of microbes that possess resistance genes, and in most of the cases, the resistance is induced through plasmids [75,76]. The recent studies on plant microbiomes have highlighted the increasing risk of plasmid-related resistant genes associated with the microbes. The application of fertilizers containing waste from animal and water sources can drastically enrich the plant’s beneficial microbes, leading to the transfer of the resistant genes. Even though it was well known that the plasmid transfer occurs because of cross-contamination and the application of animal manure, it has been a question of debate about the transmission of plasmids among soil microbiomes. The study by [77] demonstrated that changes in soil conditions, such as temperature fluctuations and moisture levels, can create stress for microbes, promoting horizontal gene transfer, and RP4 carrying antibiotic-resistant plasmid transmission occurs through horizontal gene transfer from the donor microbe to the soil microbiome and, eventually, will be carried by plant endophytic microbes.

### 5.2. Plasmids in Plant Beneficial Microbes

A diverse plant microbiome inhabits the plant, defining the plant growth and development, and studies have been focusing more on the role of the metagenome and screening of plant growth-promoting microbes [30]. The role of plant microbial plasmids is an added benefit for the plant–microbe interaction kinetics and plays a vital role in providing significant advantage in adverse environmental conditions for the survival of plants. Abiotic stress is a major factor that affects the growth and development of plants, and the changing environmental conditions exert pressure, but the mitigating effects against this adverse condition are regulated through the microbiome that carries specific plasmids. Plasmids carry specific genes that can improve nutrient acquisition and phytohormone production, mitigating the effect of abiotic stresses and improving the microbial diversity [78]. Plasmids can respond to environmental stimuli, and thus, they can be crucial, as they can expand the overall metabolic pathway of the plant by improving the nutrient acquisition [79]. The iron absorption facilitated by lncP-1β plasmid enabled the *Burkholderia terrae* microbial strain to improve its fitness when iron-deficient and improve iron absorption [80]. The plasmid PhB44 carrying this gene will also be able to transfer the plasmid to other related microbes, depending on the environmental stimuli lacking iron deficiency [81]. The classic example of nitrogen fixation in plants is a direct result of the influence of the nod operon, which is activated through the flavonoids produced through plants [82]. The host selection of the microbiome is triggered through the chemicals being secreted through the roots, and microbes have evolved to have specialized plasmids like pCSA2 *Cronobacter sakazakii* [83] and S499 *Bacillus amylo-liquefaciens* plasmids [84]. The genes associated with these plasmids have diverse mechanisms, including motility, adhesion, and biofilm-forming capabilities, that help the host to construct a selective microbial diversity. Rhizosphere-associated plant microbial plasmids are also hot spots for the degradation of pollutants, including phenols and toluene [85].

### 5.3. Plasmids Associated with Abiotic Stress

Plants are constantly being influenced by changes in the environment, and this will have a significant impact on the growth of plants [65]. The role of plasmids in abiotic stress provides valuable insight into the role of plasmids in response to abiotic stress. Drought stress is a major abiotic stress that influences the growth and development of the plant, and *Rhizobium leguminosarum bv. trifolii* W14-2 carries four plasmids that vary in size. Clover rhizobia strains without plasmids had lower survival rates under heat and drought stress in soil compared to those with plasmids. Plasmids might contain genes that help with stress tolerance, such as those involved in producing compatible solutes or repairing DNA damage, aiding the bacteria’s survival in harsh conditions [86,87]. These plasmids with a smaller molecular weight of 260 MDa and 350 MDa improved the survival efficiency of the microbes in the soil, and it clearly indicates the role of plasmids in drought tolerance [88]. Salt stress is a serious concern, and more land area is becoming unsuitable due to increase salinization, but the role of plasmids with ion salt-resistant bacteria can be a promising alternative. pSH1418 and pSH1451 plasmids are the two sets of plasmids when transformed to an *E. coli* system shown to have improved resistance against salt stresses [89]. Certain bacteria can not only survive and thrive in high salt environments but also play crucial roles in wastewater treatment and soil bioremediation by breaking down pollutants and organic matter [90]. The natural variant of this plasmid was found in *Bacillus pumilus*, and the genes coding was found to have a significant influence on the salt tolerance, especially by transporting aspartate for the regulation of osmotolerance. Heavy metal contamination can be another plant growth-limiting factor, and the role of microbes is clear from the heavy metal resistance genes associated with plasmids in resilient microbes. Plasmidome analysis of the microbes isolated from a coal mine was found to carry plasmids that process heavy metal resistance to a range of heavy metals, including cadmium, mercury, zinc, and arsenic [91].

## 6. Characterization of the Plasmidome

Recent studies have highlighted the significant role of the plasmidome in mediating plant responses to various abiotic stresses, including plasma exposure (see Figure 3b) [70]. Plasma can induce oxidative stress in plant cells, leading to a cascade of physiological and molecular responses. Additionally, the plant microbiome, which consists of diverse microbial communities associated with different plant tissues, interacts with the plasmidome to influence plant health and stress resilience. Studies have shown that plasma exposure can lead to shifts in the composition and function of the plant microbiome, potentially enhancing the plant’s ability to cope with stress [92]. These studies provide valuable insights into the mechanisms underlying plant stress responses and offer potential strategies for improving crop resilience and productivity in the face of environmental challenges [93,94]. Advanced sequencing strategies can be employed to elucidate the genetic variations induced by plasma treatment. High-throughput sequencing technologies, such as Illumina and PacBio, can be utilized to capture a comprehensive profile of plasmidome variations [95]. Illumina sequencing provided high accuracy and depth, enabling the detection of single-nucleotide polymorphisms (SNPs) and small insertions or deletions (indels) with precision. Complementarily, PacBio sequencing offered long-read capabilities, crucial for identifying larger structural variations and complex rearrangements within the plasmidome [96]. The integration of these sequencing platforms facilitated a robust comparative analysis, revealing significant shifts in the plasmid composition and abundance post-plasma exposure. Bioinformatics tools, including variant calling and genome assembly algorithms, were pivotal in interpreting the sequencing data, highlighting the dynamic nature of the plasmidome responses to plasma-induced stress in plants. Additionally, the study employed metagenomic approaches to analyze the total plasmid populations, providing insights into the ecological impacts and evolutionary significance of plasmidome variations [96].

## 7. Transcriptional Response of Microbiomes to Plasma Exposure

With recent advancements in plasma technology in different applications, such as sterilization [97,98], environmental water and soil remediation [99], and medicine [100,101,102], in step with progressions, exploring the interaction between microbiomes and plasma treatment is an emerging area of study. NTP creates an oxidative environment that significantly affects the viability and functioning of microbial communities [103,104]. Plasma-induced oxidative stress triggers a cascade of transcriptional responses as microorganisms attempt to alleviate damage and adapt to these hostile conditions [105]. Recent developments in transcriptomic analyses, such as RNA sequencing (RNA-seq), have revealed that plasma exposure induces significant changes in the expression of genes related to oxidative stress, metabolism, and DNA repair pathways [103,105]. Notably, microbial genes involved in detoxifying reactive oxygen species (ROS), such as those encoding catalases, peroxidases, and superoxide dismutases, are consistently upregulated following plasma treatment [106]. These enzymes play a crucial role in neutralizing ROS and protecting cells from oxidative damage, highlighting that plasma treatment can directly influence the stress response mechanisms. The microbial survival strategies in plasma-altered environments can be applied to develop plant stress resilience mechanisms by utilizing the plant-beneficial microbes [107].

Additionally, plasma exposure also impacts the metabolic activities of microbial communities. Transcriptional profiling studies have shown that genes associated with central metabolic pathways, including glycolysis, the tricarboxylic acid (TCA) cycle, and oxidative phosphorylation, tend to be downregulated in plasma-treated microbiomes [105]. The low expression of central metabolic pathways might indicate a possible metabolic slowdown or energy conservation strategy as the cells shift their focus toward survival under oxidative stress rather than growth or replication [105,108]. Suppressing these key metabolic processes may result in reduced ATP production, a slower growth rate, and a more energy-efficient state, which helps microbes survive adverse plasma conditions. In addition to metabolic shifts, plasma-induced DNA damage is another significant challenge for microbial communities [109]. Previous findings have documented the reduction of DNA damage in response to plasma treatment [110]. Low-temperature plasma treatment leads to an initial wave of DNA damage. In response, cells activate DNA repair mechanisms. The proteins involved in repair, such as PARP1 and XRCC1, exhibit increased expression followed by a decrease in markers of DNA damage [111,112]. The minimal damage of the DNA might indicate the possibility of activating DNA repair systems to maintain the genomic integrity [109,113]. These transcriptional changes offer valuable insights into the regulatory mechanism of microbes to adapt to plasma conditions, presenting opportunities for targeted applications (see Figure 3c). It can be proposed that plasma treatment could enhance the stress tolerance of plant-beneficial culturable microbes, enabling them to support plants more effectively under adverse conditions [114].

## 8. Metabolomic Shifts in Plants Induced by Plasma Treatment

The NTP treatment has been shown to induce significant physiological and biochemical changes in plants, particularly through its effects on metabolism [11]. Cold plasma can generate a highly reactive environment that interacts with plant tissues, leading to shifts in both primary and secondary metabolites [11,115,116]. These metabolite changes can be used in various fields, particularly in agriculture, where plasma technology can help enhance plant resilience to environmental stressors. One of the most well-documented effects of plasma treatment is the regulation of antioxidant metabolites, including phenolic acids, flavonoids, and ascorbic acid [117]. Phenolic compounds, such as caffeic and ferulic acid, are known for their roles in neutralizing free radicals, while flavonoids like quercetin and kaempferol provide both antioxidative and anti-inflammatory benefits [118,119]. These compounds are synthesized through the phenylpropanoid pathway, which is often stimulated in response to plasma-induced oxidative stress [117]. Ascorbic acid, another critical antioxidant, acts as a direct ROS scavenger and regenerates other antioxidants such as α-tocopherol and glutathione [120]. The metabolites generated during plasma exposure induce a robust defense system that detoxifies ROS and stabilizes cellular structures, resulting in reduced oxidative damage. The increased production of these antioxidants can be exploited to increase the plant’s resilience to environmental stresses, contributing to improved growth and development under suboptimal conditions [121]. These findings suggest that plasma treatment could be utilized to increase crop resilience to biotic and abiotic stresses.

Plasma treatment can also affect nitrogen metabolism through the upregulation of nitrogen-containing compounds such as amino acids, including glutamine and proline [116]. Proline is a well-known osmoprotectant and ROS scavenger that helps plants cope with oxidative stress. It stabilizes proteins and membranes and maintains the cellular redox balance, thereby inducing a protective effect [122]. This reallocation of nitrogen resources in response to plasma-induced stress highlights the plant’s ability to modulate its metabolic priorities under challenging conditions. Like nitrogen metabolism, carbohydrate metabolism is also known to be impacted by plasma exposure [123,124]. These sugars are vital for energy production, osmotic regulation, and stress signaling in plants. Plasma-induced oxidative stress possibly triggers changes in these processes as part of an adaptive mechanism to balance energy demands and maintain cellular homeostasis under stress. Moreover, plasma treatment has been shown to stimulate secondary metabolic pathways, leading to the increased production of bioactive compounds like alkaloids, terpenoids, and phenolic acids. These metabolites enhance plant defense and offer potential applications in improving the nutritional and medicinal value of crops [116,117,118,119,125]. Plasma-induced metabolomic shifts in plants demonstrate a complex, multifaceted response to oxidative stress, affecting both primary and secondary metabolism. These metabolic adjustments enable plants to adapt to environmental stressors, highlighting the potential of plasma treatment as a tool for improving plant health and productivity in agriculture (see Figure 3d).

## 9. Future Directions

### Plasma as a Tool for Engineering Beneficial Plant–Microbe Interactions

The application of plasma technology in agriculture, particularly for engineering beneficial plant–microbe interactions, presents a promising avenue for enhancing plant health and productivity. Plasma treatments have demonstrated significant potential in promoting the activity of plant growth-promoting bacteria (PGPB), controlling plant pathogens, and improving soil health. These benefits contribute to a more sustainable and eco-friendly approach to agriculture, reducing the reliance on chemical pesticides and fertilizers [33,49,55]. Moreover, the integration of plasma technology into space research offers exciting possibilities for supporting plant growth in extraterrestrial environments. As humanity prepares for long-term space missions and the potential colonization of other planets, the ability to cultivate healthy crops in space becomes crucial. Plasma treatments can enhance plant resilience and microbial management in controlled space habitats, ensuring the sustainability of life support systems [51,126,127]. Plasma treatment in agriculture has wider applications but comes with certain advantages and disadvantages, as discussed in Table 1. Future research should focus on optimizing plasma treatment parameters to maximize benefits while minimizing any potential adverse effects on beneficial microbes. Developing portable and efficient plasma generation systems will also be essential for practical applications in space missions. By continuing to explore and refine these technologies, we can unlock new potentials for terrestrial and extraterrestrial agriculture, paving the way for a more sustainable future.

## Figures and Tables

**Table 1:** The advantages and disadvantages of plasma treatment on plants.

Treatment Condition	Advantages	Disadvantages	References
Seed germination and treatment	Germination rates increase Surface sterilization of seeds Improve water and nutrient uptake	Optimization step can be time and labor intensive High infrastructure and instrument cost	[128,129]
Preservation	Reduced microbial contamination Long term storage Higher food safety standards	Extensive study to determine the effect of plasma on food texture and quality Higher energy consumption for plasma treatment	[130]
Soil quality	Enhance soil nutrient quality Improve soil microbiome	Technical limitations to cover a larger area	[131]
Plant growth	Improves plant growth and development	Energy intensive processes that demand further research to optimize treatment conditions	[132]
